# Recombinant chromosome with partial 14 q trisomy due to maternal pericentric inversion

**DOI:** 10.1186/s13039-015-0195-7

**Published:** 2015-11-21

**Authors:** Hande Küçük Kurtulgan, Leyla Özer, Malik Ejder Yıldırım, Evrim Ünsal, Süleyman Aktuna, Volkan Baltacı, Nejmiye Akkuş, İlhan Sezgin

**Affiliations:** Cumhuriyet University School of Medicine Division of Medical Genetics, Sivas, Turkey; Mikrogen Genetic Diagnosis Center, Cinnah Street 47/1 Çankaya, Ankara, Turkey; Division of Histology and Embryology, Yeni Yüzyıl University School of Medicine, İstanbul, Turkey; Medicine Division of Medical Biology and Genetics, Yeni Yüzyıl University School of Medicine, İstanbul, Turkey

**Keywords:** Pericentric inversion of chromosome 14, 14q duplication, Microcephaly, Cardiac defects

## Abstract

**Background:**

14q duplications caused by parental pericentric inversion of chromosome 14 are rarely reported and no clear genotype-phenotype correlation has been determined yet.

**Case Presentation:**

Here we reported a 7 years old female patient with recombinant chromosome characterized by 14 q duplication and originated from maternal pericentric inversion of chromosome 14. Principal clinical findings of the child include developmental delay, microcephaly, hypertelorism, low set ears, clinodactyly of fifth fingers, hypotonia, telecanthus and cardiac malformation.

**Conclusions:**

Her final karyotype was 46,XX,rec(14)dup(14q)inv(14)(p11.2q24)mat,arr14q24.1-qter(64,800,000-108,350,000 bp)x3.

## Background

Pericentric inversions are intrachromosomal rearrangements which have one break in the short arm and one in the long arm of the chromosomes. Pericentric inversions (excluding variant forms) have a frequency range about from 0,12 % to % 0,7 [1]. The clinical importance of pericentric inversion arises from increased risk of generation of recombinant gametes that may lead to abnormal pregnancy. Pericentric inversion carriers have risks for affected child or habitual abortions due to duplication deficiencies caused by crossing over during prophase of meiosis I within the inverted segment(1). Chromosome 14 often involves in chromosomal rearrangements but pericentric inversions of chromosome 14 are rare events [2, 3]. Partial trisomy 14q resulting from parenteral pericentric inversions have been rarely described in literature. Partial trisomy 14q has been showed frequent features as low birth weight, developmental delay, failure to thrive, mental retardation, hypotonia, microcephaly, wide fontanelles, hypertelorism, pinched nose, prominent over lip, ear anomalies, finger anomalies, dacryostenosis, fish mouth, telecanthus and congenital heart defects. Here we report a family with a mother carrying pericentric inversion and her daughter with recombinant chromosome 14.

## Case presentation

The proband (pedigree number III.3) was 7 months years old female patient and the third child of non-consanguineous, healthy parents who have three children. The female patient was referred to our genetic diagnosis center because of dysmorphic features and growth retardation.

**Fig. 1:**
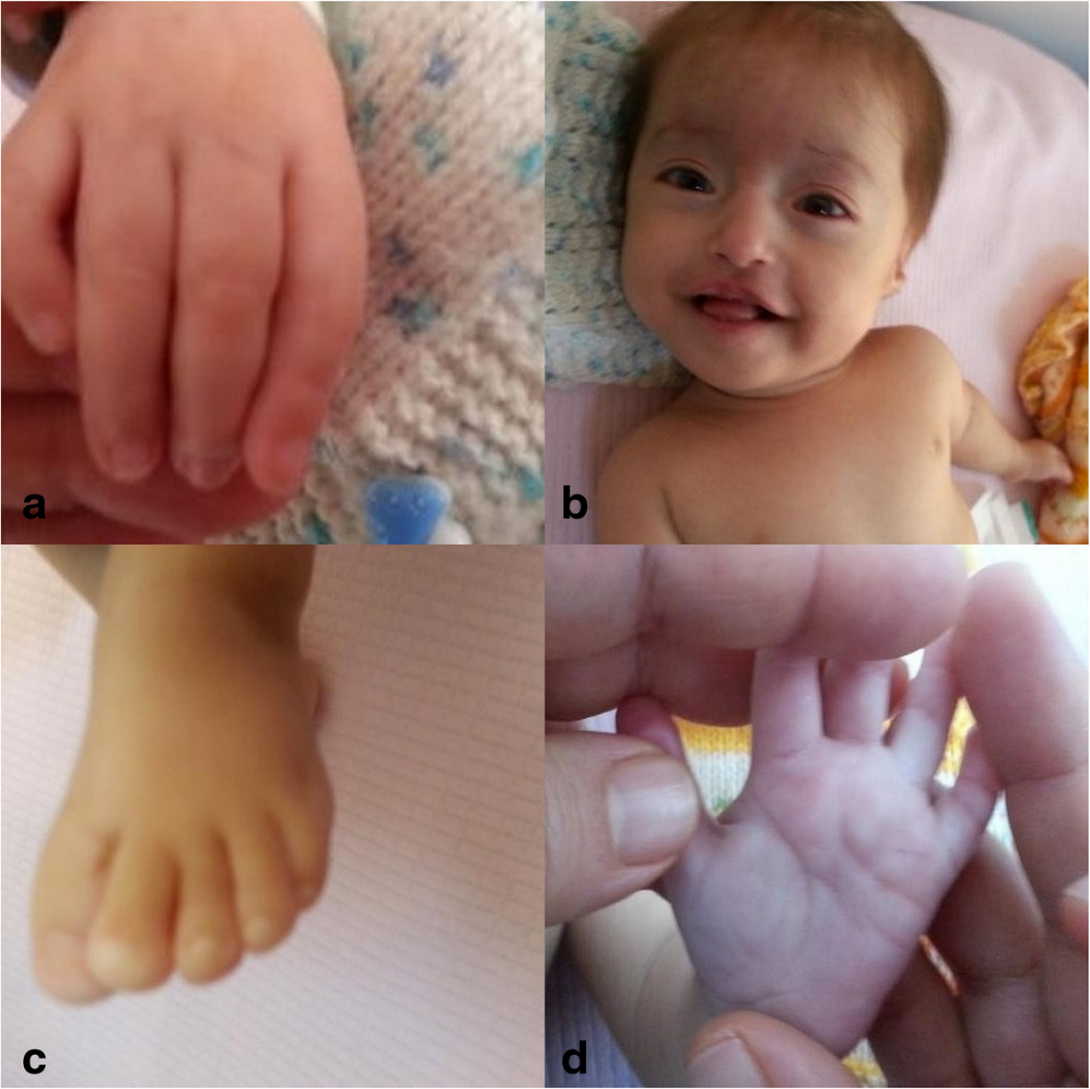
Clinical features of proband. **a.** Clinodactyly of fifth finger of hand of our patient **b.** Telechantus, pinchnose, dysplastic ears **c**. Mild syndactyly between 2. and 3.fingers of feet **d**. Sydney line in palm of hand

Physical examination revealed a height of 58 cm (below 3 % percentile) and weight of 4300g (below 3 % percentile) and growth retardation was noted. The measurement of head circumference was 39 cm (below 3 % percentile) and microcephaly was found. Dysmorphology assessment revealed frontal bossing, telecanthus, down slanting and short palpebral fissures, dacryostenosis, hypertelorism, depressed nasal root, pinch nose, fish mouth, low set ears, syndney line on hands bilaterally, protruding tongue, clinodactyly of fifth fingers, syndactyly of second and third toes bilaterally, anteverted nostrils (Fig. 1). Hemangioma was seen on forehead. Ecocardiography showed that she had ventricular septal defect. Hypotonia was noted.

Chromosome analysis was performed from lymphocyte cultures with standart G-banding methods. G banded analysis of proband, parents and siblings had 450–550 band resolution. CGH array analysis was prepared according to manufacturer's instructions (Illumina, 24 sure CGH array). Array data was analyzed by Blue Fuse Multi v3.0 software system.

**Fig. 2:**
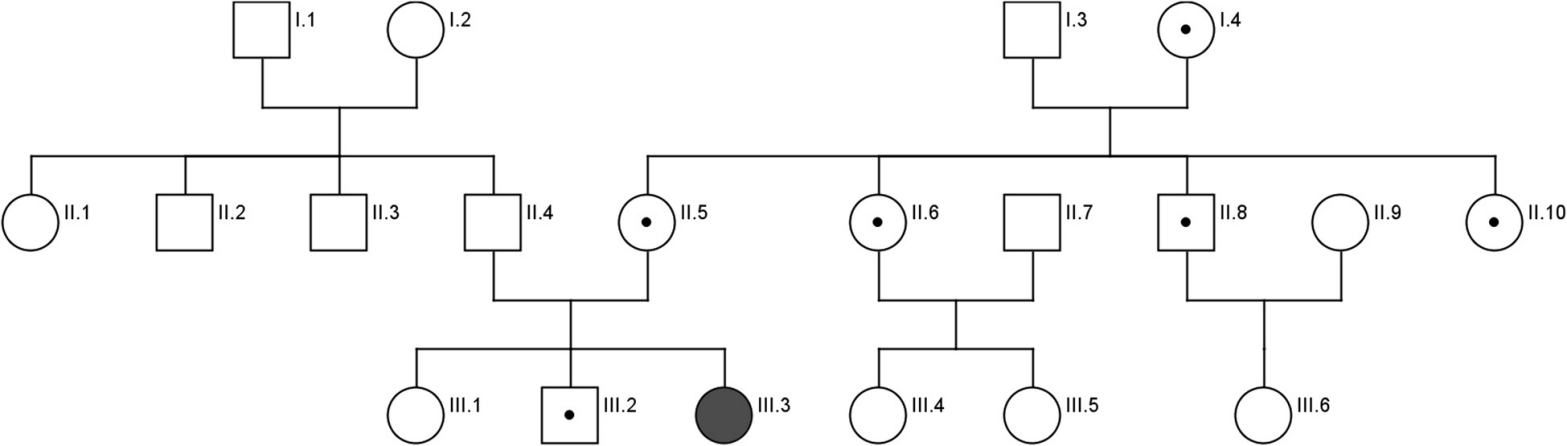
Pedigree analysis of family. Proband (46,XX,rec(14)dup(14q)inv(14)(p11.2q24) are shown as filled black symbols (III.3) whereas inversion carriers (46,XX,inv(14)(p11.2q24) are shown as dot-filled symbols (III.2, II.5, II.6, II.8,II.10,I.4). Males are shown as squares and females as circles

**Fig. 3:**
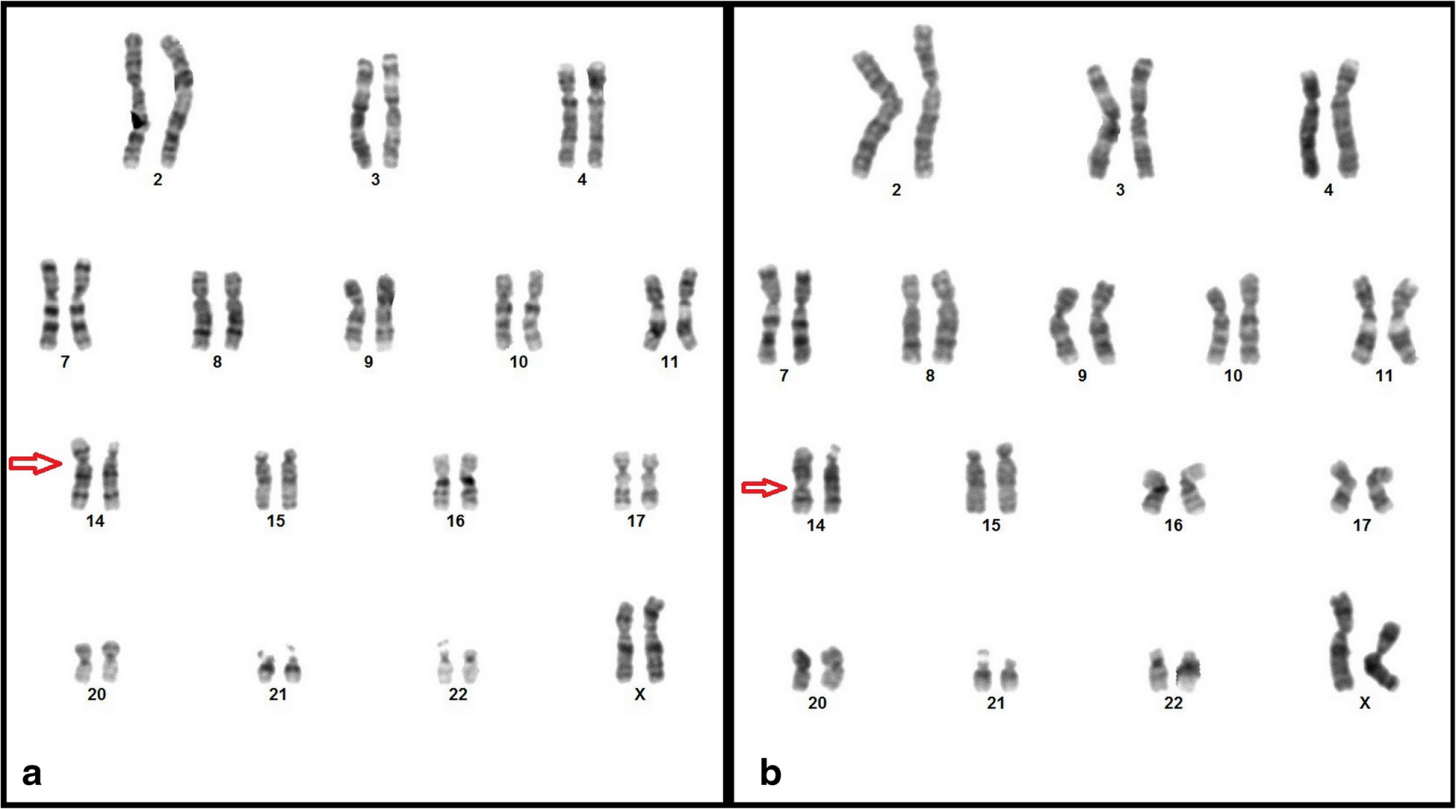
Cytogenetic analysis. **a.** Karyotype analysis of proband with derivative chromosome 14 (46,XX,rec(14)dup(14q)inv(14)(p11.2q24)mat). **b.** Karyotype analysis of proband's mother with pericentric inversion of chromosome 14 (46,XX,inv(14)(p11.2q24))

We performed G-banded chromosomal analysis of proband (Fig. 2. pedigree number III.3) and it revealed 46,XX,add(14)(p10) karyotype (additional material on the short arm of chromosome 14) in 20 analyzed metaphases (Fig. 3a). To determine the origin of addition material on chromosome 14 we performed parental karyotype analysis. Proband’s father had normal 46,XY karyotype (Fig. 2. pedigree number II.4) yet mother’s chromosome analysis revealed a pericentric inversion of chromosome 14 (Fig. 2. pedigree number II.5). Her karyotype was 46,XX,inv(14)(p11.2q24) (Fig. 3b). Also we performed karyotype analysis for proband’s siblings. Her sister’s karyotype (Fig. 2. pedigree number III.1) was normal but her brother’s karyotype (Fig. 2. pedigree number III.2) was 46,XY,inv(14)(p11.2q24). He carried the same pericentric inversion of chromosome 14. We detected that the mother, mother's sisters and the brother are also carrier for the same pericentric inversion of chromosome 14 (Fig. 2).

**Fig. 4:**
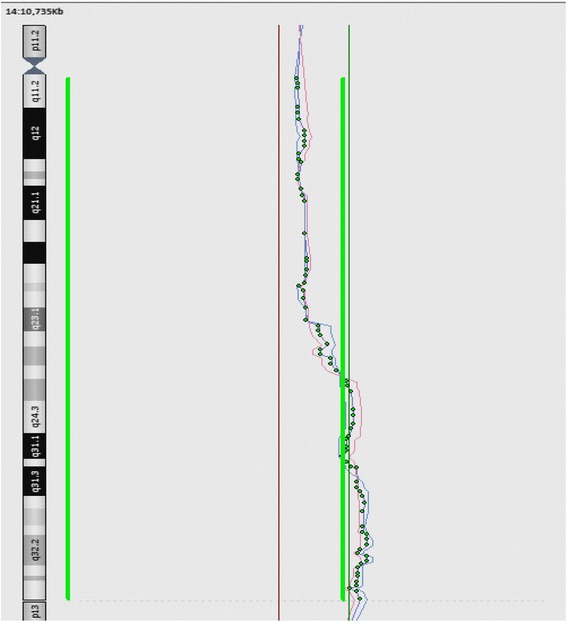
CGH array. CGH array profile of derivative chromosome 14 indicating a duplicated region of chromosome 14q

CGH array analysis of proband showed a segment duplicated between 14q24.1qtel region with approximately 43.5 Mb (breakpoints: 64,800,000–108,350,000). (Fig. 4). According to ISCN nomenclature writing the proband's karyotype was 46,XX,rec(14)dup(14q)inv(14)(p11.2q24)mat, arr14q24.1qtel(64,800,000–108,350,000)x3 .

## Discussion

Pericentric inversions are structural rearrangement with two-break event which both breaks are on either side of the centromere (1, 4). There is an tendency homolog chromosome regions to pair during prophase of meiosis-I which indicates that pairing of inverted segments, in an inversion heterozygote, leads to the information of an inversion loop. As a consequence, pericentric inversion carriers have an increased risk of generating an offspring with a recombination chromosome presenting a deletion and (or) a duplication of chromosomal distal segment [3–5]. An unbalanced recombinant chromosome will rise largely depends on size of the inverted segment during meiosis. The size of inversion is related with meiotic crossover event occurring within the inverted segment. This event will give result depend of the amount of duplicated and deleted material present in the resulting recombinant chromosomes [6].

In our report the proband had recombinant chromosome 14 which showed the duplication of distal part of chromosome 14 (partial 14q duplication). This problem may arise from duplication of 14q in crossover of meiosis 1 in her mother. The reason of the clinical findings of our patient is arisen from partial trisomy 14q. Partial 14 q duplications have resulted from parental translocations and pericentric inversions. 14 q duplications have recognizable clinical features but some important phenotypic variations as cardiovascular anomalies, urogenital anomalies can be seen. These variations might be related with length of the duplicated segment. Several cases of partial duplications of 14q have been reported and according to these reports phenotypic features of distal 14q duplications are less severe than proximal 14q duplications. Gene density in chromosome 14 might be high and the change of a large amount of genes could explain the clinical severity of proximal 14 q duplications [7]. Most of the partial 14 q duplication cases have following clinical features; low birth weight, mental retardation, hypotonia, microcephaly, wide fontanelle, hypertelorism, dysmorphic nose, prominent overlip, ear anomalies, finger anomalies, congenital heart defects [2, 3, 7–10]. Compatible with these reports our patient has similar clinical features.

Breakpoints, size of segment duplicated, and genes involved are variable among cases with this chromosome rearrangement. According to DECIPHER database (https://decipher.sanger.ac.uk) the 14q24.1-qter region of chromosome 14 involves more than 500 genes. At DECIPHER database a patient (250364) with a duplication at chromosome 14 of 20.91 Mb (break points: 84,783,824–105,689,917 bp) and a proximal deletion of 1.0 Mb has hypoplasia of corpus callosum, low-set ears, atrial septal defect, facial abnormalities and submucous cleft hard palate. The other case (2587) which was described at DECIPHER has a duplication of chromosome 14 of 7.61 Mb (break points: 99,671,787–107,284,480 bp). The findings of patient described included: brachydactyly syndrome, frontal bossing, hypertelorism, hyperextensibility of the finger joints, intellectual disability, prenatal short stature and wide mouth. The other DECIPHER case (1593) with a duplication at chromosome 14 have abnormality of the heart, abnormality of the pinna, abnormality of the tarsal bones, abnormality of the upper respiratory tract, blepharophimosis, blue sclerae, feeding difficulties in infancy, hypertelorism, intellectual disability, microcephaly, micrognathia, muscular hypotonia, patent ductus arteriosus, recurrent infections, seizures, short stature, single transverse palmar crease, sleep disturbance, wide nasal bridge. Our patient's clinical features match with the reported 14 q duplication cases in DECIPHER (https://decipher.sanger.ac.uk) and literatures [2, 3, 7–11]. Specifically clinical findings of our case are similar with 14 q duplication case of Kaiser [2]. Microcephaly, hypertelorism, dacryostenosis, hypotonia, dysmorphic nose was reported in Kaiser’s study. The breakpoints of parental pericentric inversion(14p11-q24) and the size of duplicated region (14q24) was similar to our case [2]. Brachydactyly, clinodactyly, hypertelorism, dysmorphic nose findings are also same with Thiel’s case however the duplication of chromosome 14q was distal part of chromosome 14(14q32.2-qter) [10]. Very similar findings of all above cases might be addressed to this loci.

Chromosome 14 contains an imprinted gene cluster at 14q32 which is regulated by the differentially methylated regions (DMRs). Chromosome 14 UPD (uniparental disomy) cases have different phenotypes depending on the parent of origin [3, 12]. The patient herein reported has some similar findings UPD cases but most of the clinical features of our patient do not match with maternal or paternal UPD chromosome 14 cases.

## Conclusions

Pericentric inversion of chromosome 14 is rarely seen. Up to date there are few cases reported which have recombinant chromosome 14 due to parental pericentric inversion of chromosome 14 [2, 3, 7–10]. Reporting patients with recombinant chromosome 14 will be helpful to characterize the typical clinical features for this chromosomal rearrangement. Evaluation of more cases with duplication of chromosome 14q24-qter region and to make linkage analyses of informative cases can clarify the phenotype-genotype correlation of this region. This study gives a new data about clinical features of partial 14q trisomy and CGH array analysis showed the visualization of the genes involved in the duplicated segment. Genetic counseling of pericentric inversions is very important and complicated because of having increased risk for generation of recombinant chromosomes so such reports also will be helpful for counseling.

## Consent

Written informed consent was obtained from the parents of the patient for publication of this case report and any accompanying images. A copy of the written consent is available for review by the Editor-in-Chief of this journal.

## Abbreviations

Array-CGH: Microarray-based comparative genomic hybridization
DECIPHER: DatabasE of Chromosomal Imbalance and Phenotype in Humans using Ensembl Resources
